# Antibodies to neutralising epitopes synergistically block the interaction of the receptor‐binding domain of SARS‐CoV‐2 to ACE 2

**DOI:** 10.1002/cti2.1260

**Published:** 2021-03-07

**Authors:** Manisha Pandey, Victoria Ozberk, Sharareh Eskandari, Ahmed O Shalash, Michael A Joyce, Holly A Saffran, Christopher J Day, Ailin Lepletier, Belinda L Spillings, Jamie‐Lee Mills, Ainslie Calcutt, Fan Fan, James T Williams, Danielle I Stanisic, Laetitia Hattingh, John Gerrard, Mariusz Skwarczynski, Johnson Mak, Michael P Jennings, Istvan Toth, D Lorne Tyrrell, Michael F Good

**Affiliations:** ^1^ Institute for Glycomics Griffith University Gold Coast QLD Australia; ^2^ University of Queensland Brisbane QLD Australia; ^3^ University of Alberta Edmonton AB Canada; ^4^ Olymvax Biopharmaceuticals Chengdu China; ^5^ Gold Coast Hospital and Health Service Gold Coast QLD Australia

**Keywords:** ACE‐2, memory B cells, peptide epitopes, receptor‐binding domain, SARS‐CoV‐2, tetramer staining, vaccine

## Abstract

**Objectives:**

A major COVID‐19 vaccine strategy is to induce antibodies that prevent interaction between the Spike protein's receptor‐binding domain (RBD) and angiotensin‐converting enzyme 2 (ACE2). These vaccines will also induce T‐cell responses. However, concerns were raised that aberrant vaccine‐induced immune responses may exacerbate disease. We aimed to identify minimal epitopes on the RBD that would induce antibody responses that block the interaction of the RBD and ACE2 as a strategy leading to an effective vaccine with reduced risk of inducing immunopathology.

**Methods:**

We procured a series of overlapping 20‐amino acid peptides spanning the RBD and asked which were recognised by plasma from COVID‐19 convalescent patients. Identified epitopes were conjugated to diphtheria‐toxoid and used to vaccinate mice. Immune sera were tested for binding to the RBD and for their ability to block the interaction of the RBD and ACE2.

**Results:**

Seven putative vaccine epitopes were identified. Memory B‐cells (MBCs) specific for one of the epitopes were identified in the blood of convalescent patients. When used to vaccinate mice, six induced antibodies that bound recRBD and three induced antibodies that could partially block the interaction of the RBD and ACE2. However, when the sera were combined in pairs, we observed significantly enhanced inhibition of binding of RBD to ACE2. Two of the peptides were located in the main regions of the RBD known to contact ACE2. Of significant importance to vaccine development, two of the peptides were in regions that are invariant in the UK and South African strains.

**Conclusion:**

COVID‐19 convalescent patients have SARS‐CoV‐2‐specific antibodies and MBCs, the specificities of which can be defined with short peptides. Epitope‐specific antibodies synergistically block RBD–ACE2 interaction.

## Graphical abstract

We identified SARS‐COV‐2 receptor‐binding domain (RBD)‐specific antibodies in convalescent patients, specificities of which could be defined with short peptides. These peptides when used as immunogens, induced neutralising antibodies which synergistically blocked RBD and ACE 2 interaction. Our approach will aid the design of a vaccine containing minimal antigenic material, thus improving the vaccine's safety profile.

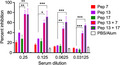

## Introduction

SARS‐CoV‐2 first entered the human population in December 2019. Currently, over 150 vaccines to prevent infection with this coronavirus are in various stages of development, representing the most rapid response by academia, industry and governments to any pathogen. The vaccine candidates for SARS‐CoV‐2 that have been described to date consist of vectored^1^, ^2^, ^3^, ^4^ and recombinant constructs^5^ containing the gene or gene product for the Spike protein of the virus, and attenuated vaccines.^6^ As of early 2021, 68 vaccines are in clinical trials and 20 are in Phase III trialling. Ten are either fully approved or approved for limited use. In pre‐clinical and early clinical studies, vaccines were shown to induce antibody responses that neutralised the virus *in vitro*. Some of the vaccines were shown to induce protection in animal models.^7^ van Doremalen et *al*. showed that an adenovirus vectored Spike protein vaccine protected monkeys from pneumonia, but virus could still be found in other tissues in the vaccinated group.^8^ Preliminary results have been published for some of the clinical trials and showed protection against symptomatic disease of 62–95% over the first 2 months following vaccination.^9^, ^10^

While there is optimism that one or more of these vaccine candidates will be successful, there are critical issues to address. Firstly, a successful vaccine must induce a durable response of sufficient magnitude that will protect for at least 1 year, enabling a COVID‐19 vaccine to be administered in a similar schedule to vaccines for influenza. Secondly, the vaccine must have an excellent safety profile. This is particularly relevant for a vaccine against a disease for which the infection fatality rate in young people is very low: ~ 1/60 000 in those aged 5–9 years and ~ 1/300 000 for those aged 10–19 years.^11^ If herd immunity is to develop, it is critical that the vaccine protects against asymptomatic infection and blocks transmission and is administered to younger people as they are the age group amongst which most transmission is occurring.

While all currently licensed vaccines have an excellent safety profile, it cannot be assumed that a SARS‐CoV‐2 vaccine will be equally safe. There are data to suggest that antibodies to coronaviruses, in some patients, may accentuate infection. This has been demonstrated for the common cold coronaviruses, where up to 40% of re‐infections can be more severe in terms of viral load than the initial infection^12^ and in animal models for SARS‐CoV‐1, it was shown to be due to antibody‐dependent enhancement (ADE) of infection.^13^ In other studies, different SARS‐CoV‐1 vaccines resulted in lung histopathology with eosinophilic infiltrates in mice^14^ and hepatitis in ferrets.^15^ Vaccination with a vaccinia‐vectored Spike protein from a cat coronavirus (Feline Infectious Peritonitis Virus) led to low titres of neutralising antibodies but enhanced disease and early death following infection^16^ via a mechanism consistent with ADE. This mechanism may be responsible for the enhanced pathology in SARS‐CoV‐1 observed in a macaque model.^17^ ADE has also been observed with other non‐corona viruses.^18^ Wang et *al*. demonstrated that certain epitopes on the Spike protein of SARS‐CoV‐1 could induce neutralising antibodies whereas antibodies of other specificities led to enhanced infection.^19^ The rate of immunopathology following infection of SARS‐CoV‐2‐vaccinated individuals may differ with different vaccines and may not be apparent until large numbers of vaccinated people have been exposed to the virus.

CD4^+^ and CD8^+^ T‐cell responses have been defined for SARS‐CoV‐2,^20^, ^21^ and recent studies show that T cells can provide anti‐viral protection.^22^ Adoptive T‐cell transfer studies in mice showed that T cells could protect against SARS‐1.^23^ However, deep immune profiling has revealed that a subset of COVID‐19 patients who do poorly have elevated numbers of activated proliferative T cells and a relative lack of circulating T‐follicular helper cells.^24^ Thus, virus‐specific T‐cell responses, particularly in the absence of an antibody response, may be pathogenic in some patients.

Vaccine‐induced autoimmunity poses a challenge for SARS‐CoV‐2 vaccines. Over one third of immunogenic sequences in SARS‐CoV‐2 have homology to human proteins.^25^ In order to develop the safest vaccines, it is prudent to use the least amount of antigenic material required for protection. A strategy to minimise the chances of developing immune‐mediated pathology is to include in the vaccine only those epitopes that are responsible for induction of a virus‐neutralising response. Synthetic peptides can be used to map epitopes and create minimalist vaccines. They have been used to define linear epitopes as well as conformational epitopes such as those constrained by α‐helical folding of a protein.^26^ Once identified, peptide epitopes can then be ligated chemically to carrier proteins with a known safety profile and create highly immunogenic multi‐valent constructs capable of inducing defined responses.^27^ Currently, peptide vaccines are in development for various viral, bacterial and parasitic diseases.^28^, ^29^

A COVID‐19 vaccine based solely on peptides representing neutralising B‐cell epitopes could be highly immunogenic, and because epitopes were identified on the basis of antibody recognition and not T‐cell reactivity, they may be less likely to induce T‐cell‐mediated immunopathology. Vaccines containing only B‐cell epitopes have been shown to induce protective responses against other organisms and these responses are boosted by exposure to the organism resulting in prolonged vaccine‐induced immunity.^30^

The receptor‐binding domain (RBD) of the Spike protein consists of anti‐parallel β sheets and connecting α helices.^31^ This structure is very amenable to epitope mapping using synthetic peptides, providing that the peptides are of sufficient length to maintain α‐helical folding. It has been shown that peptides of 20 amino acids are of sufficient length to maintain a ⍺‐helical folding and immunogenicity.^32^ To develop a vaccine candidate that will induce a focused virus‐neutralising antibody response, we used a library of 20‐mer peptides to initially identify epitopes from the RBD of the Spike protein recognised by plasma from COVID‐19 convalescent patients. These epitopes were then used to construct synthetic immunogens which were assessed for their ability to induce virus‐neutralising antibodies.

## Results

### COVID‐19 convalescent plasma recognises recRBD and neutralises SARS‐CoV‐2 *in vitro*

**Figure 1:**
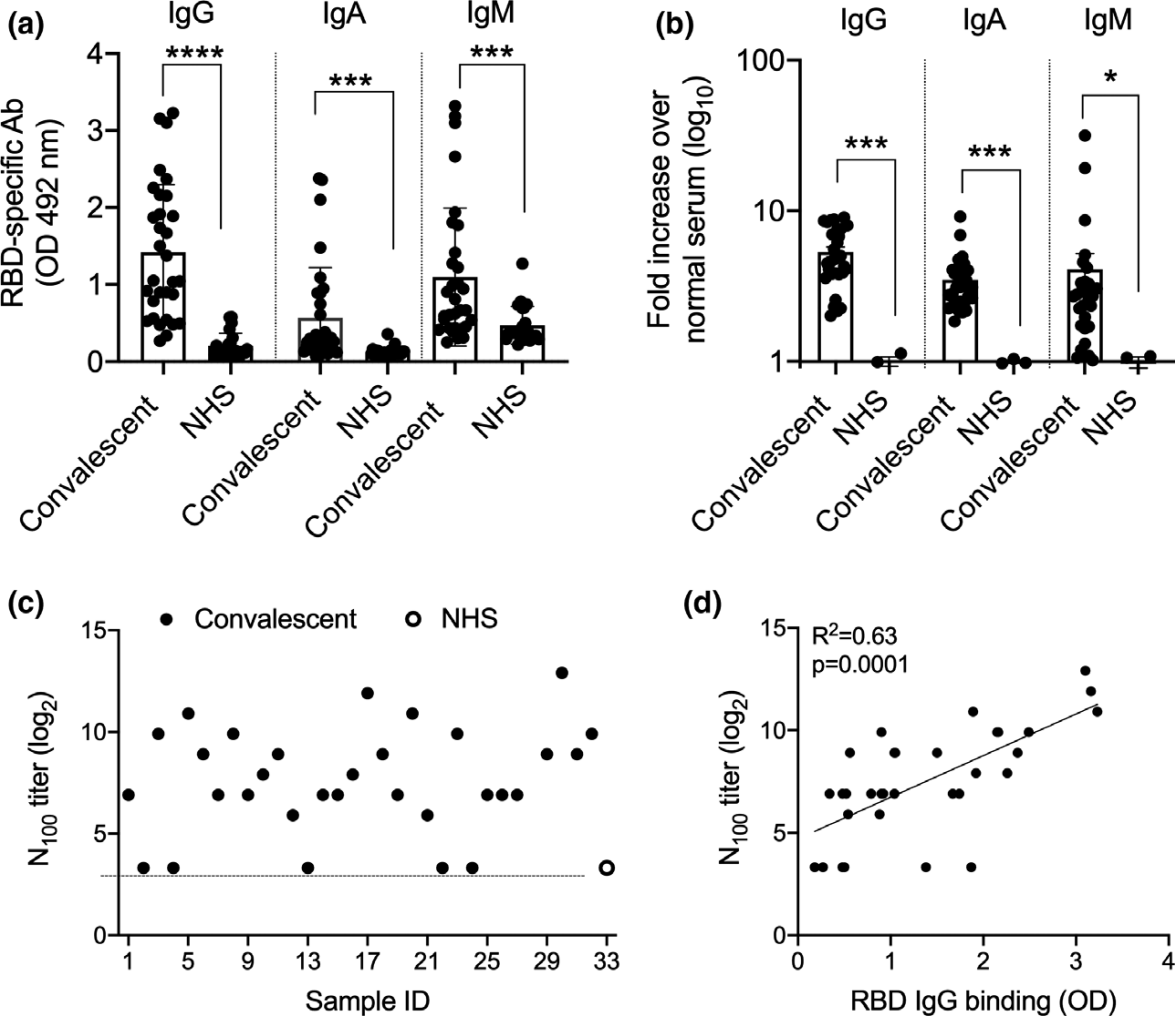
Assessment of RBD‐specific and neutralising antibodies in COVID‐19 convalescent plasma. **(a)** Antibody responses to SARS‐CoV‐2 in COVID‐19 recovered patients. CP from COVID‐19 patients were tested in ELISA for their binding to immobilised recRBD. The data are Mean ± SEM. Statistical analysis was performed using nonparametric, unpaired Mann–Whitney *U*‐tests to compare the convalescent and NHS samples. **(b)** Quantitation of IgG, IgA and IgM binding of CP to vero cells infected with SARS‐CoV‐2. Each CP was run in duplicate wells. High content imaging of the IgG, IgA and IgM staining of cells is shown. The analysis represents approximately 35 000 cells per CP. The J2 antibody was used to mark infected cells and its fluorescence intensity was used to normalise the fluorescence in the IgA, IgG and IgM channels on a per cell basis as described in the Methods. *P < 0.05, ***P < 0.001, ****P < 0.0001. **(c)** Neutralising antibody responses to SARS‐CoV‐2 in COVID‐19 recovered patients. Sample # 1–32 are convalescent plasma and ‘O’ (NHS) in the graph represents the titre of the pool of 20 serum samples. **(d)** The correlation between CP IgG binding to recRBD and neutralising antibody. Linear regressions with Pearson correlations coefficients are shown.

We used convalescent plasma (CP) from patients recovered from COVID‐19 (Supplementary table 1) and asked whether they contained antibodies that recognised recombinant RBD (recRBD). We compared 32 CP with 20 normal human serum (NHS) samples collected prior to December 2019 (pre‐COVID era) and measured RBD‐specific IgG, IgA and IgM by ELISA (Figure 1a). Most CP contained significantly greater levels of RBD‐specific antibodies of all classes than NHS. These observations were also confirmed using Surface Plasmon Resonance (SPR; Supplementary figure 1a). High content fluorescent imaging demonstrated a significantly higher binding of IgG, IgA and IgM antibodies in CP to SARS‐CoV‐2 infected Vero cells, in comparison with NHS (Figure 1b, Supplementary figure 2). We asked whether CP binding to RBD (measured by ELISA) correlated with the binding of CP to infected Vero cells (measured by fluorescent imaging). We assessed IgG, IgA and IgM separately and observed a significant correlation between RBD‐specific IgG ELISA antibodies and IgG fluorescent imaging antibodies to virus‐infected cells (Supplementary figure 3a). However, no such correlations were observed for IgA and IgM antibodies (Supplementary figure 3c and e).

We tested CP for their ability to neutralise SARS‐CoV‐2 infection of Vero cells *in vitro*. The dilution at which CP could neutralise 100 plaque‐forming units (pfu) of SARS‐CoV2 in Vero E6 cells was called the N_100_ titre. Twenty‐seven of the 32 CP that we tested had N_100_ titres between 30 and 3840 (Figure 1c). CP were also shown to inhibit the interaction between ACE2 and recRBD using SPR (Supplementary figure 1b). Neutralising activity correlated with RBD‐specific IgG, IgA and IgM levels in the CP (Figure 1d, Supplementary figure 4). Neutralising activity also correlated significantly with virus specific IgG, IgA and IgM in the CP determined using fluorescent imaging (Supplementary figure 3b, d and f).

### Identifying neutralising epitopes

**Figure 2:**
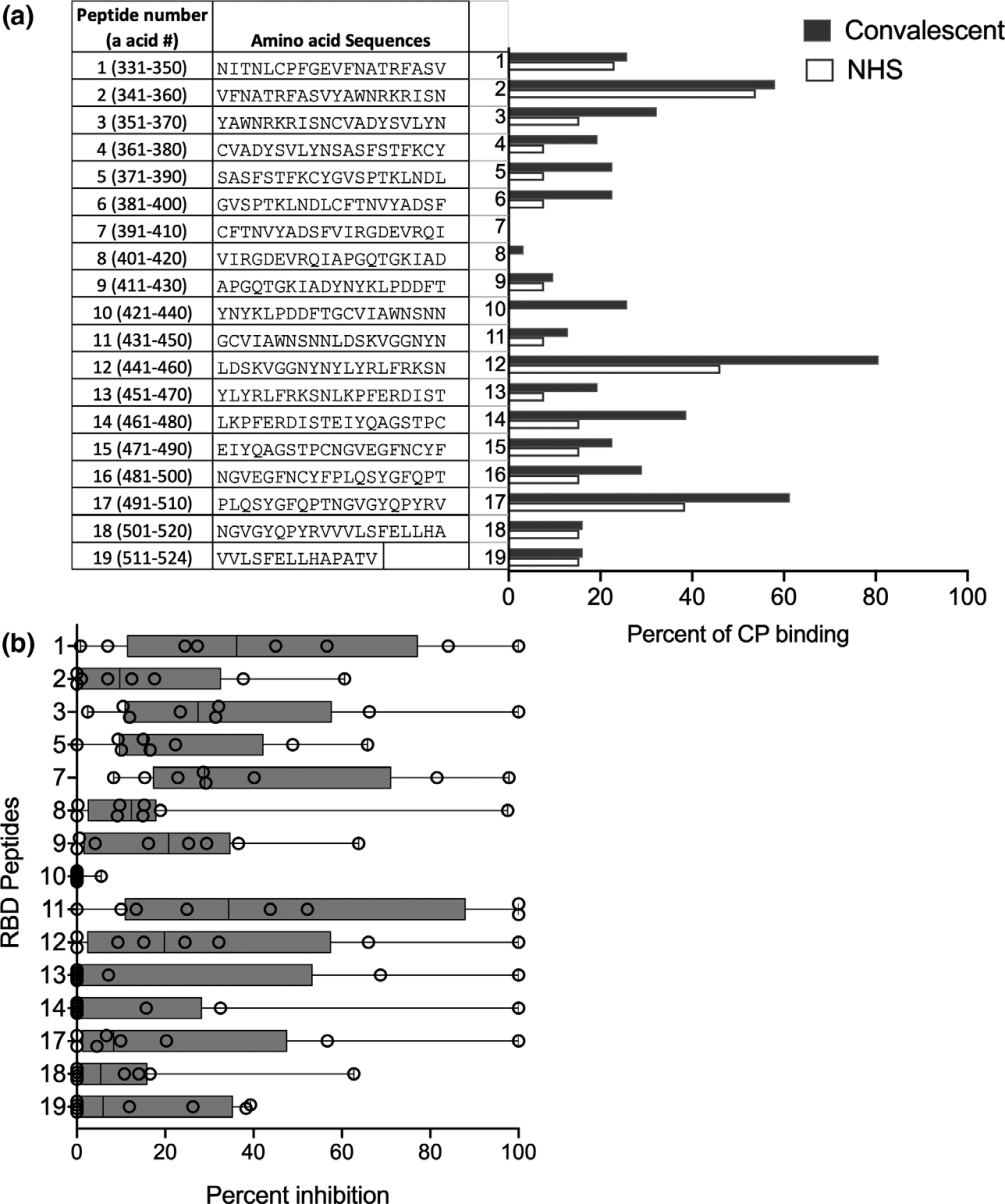
RBD peptide recognition by CP. **(a)** Sequences of overlapping peptides from the RBD peptide library and their recognition by CP. CP and NHS were tested for their binding to immobilised 20‐mer peptides from the RBD library. A total of 32 CP and 20 NHS (10 male and 10 females) were tested in ELISA and the percentages that bound to each peptide are shown. Cut‐off for a positive response is where the O.D. for each plasma sample exceeds the mean O.D. to the random peptide control by at least 2SD. **(b)** Inhibition of CP antibodies binding to immobilised RBD by specific peptides in solution. Competition ELISA was used to assess the ability of the peptides to inhibit the binding of the 8 highest titre CPs to RBD. Recombinant RBD and the random peptide were used as positive and negative controls. The % inhibition data are box and whiskers plots, with individual CP samples spanning the minimum to maximum range.

To identify epitopes within the RBD recognised by CP, we used a biotinylated peptide library of 20‐mer synthetic peptides, each overlapping by 10 amino acids and attached the peptides to an ELISA plate using streptavidin. A control 20‐mer peptide with a sequence derived using a random number generator was also tested. Both CP and NHS samples recognised RBD peptides with positivity determined by an O.D. reading exceeding the mean O.D. reading for the control peptide by > 2 S.D. Nine peptides (3–6, 10, 12–14 and 17) were recognised by a higher fraction of the CP samples than the NHS samples (Figure 2a). The peptides recognised by the highest percentage of CP were peptides 12, 17 and 2 (80%, 61% and 58% recognition, respectively). To explain the low levels (up to 20–40%) of binding observed with NHS, we investigated the sequence homology between the RBD peptides and seasonal coronavirus (*HCoV‐NL63, HCoV‐229E, HCoV‐OC43* and *HCoV‐HKU1*) proteins using BLAST search. The sequence alignment of the RBD peptides with selected top hit protein(s) from the coronaviruses demonstrated varying degrees of percent identity. In general, all peptides showed some homology with seasonal coronaviruses (Supplementary table 2). To identify epitopes that may be concealed when bound to an ELISA plate, we tested all peptides that were available in a competition ELISA using recRBD and the eight most inhibitory CP. The peptides were added to the plasma and incubated for 30 min prior to the plasma being added to the ELISA plate coated with recRBD. Inhibition by recRBD in solution was defined as 100% and by the non‐specific control peptide as 0%. We observed 10 instances where a single peptide was able to inhibit by more than 90% the binding of CP to recRBD (Figure 2b). However, in most instances a single peptide provided only partial inhibition. The assay showed that peptide 7 was seen by CP when in solution even though this peptide was not recognised when immobilised on plastic. The results from these two assays were then used to select seven peptides (1, 2, 7, 12, 13, 14 and 17) to be taken forward for immunogenicity testing.

### Identifying putative peptide vaccine candidates

**Figure 3:**
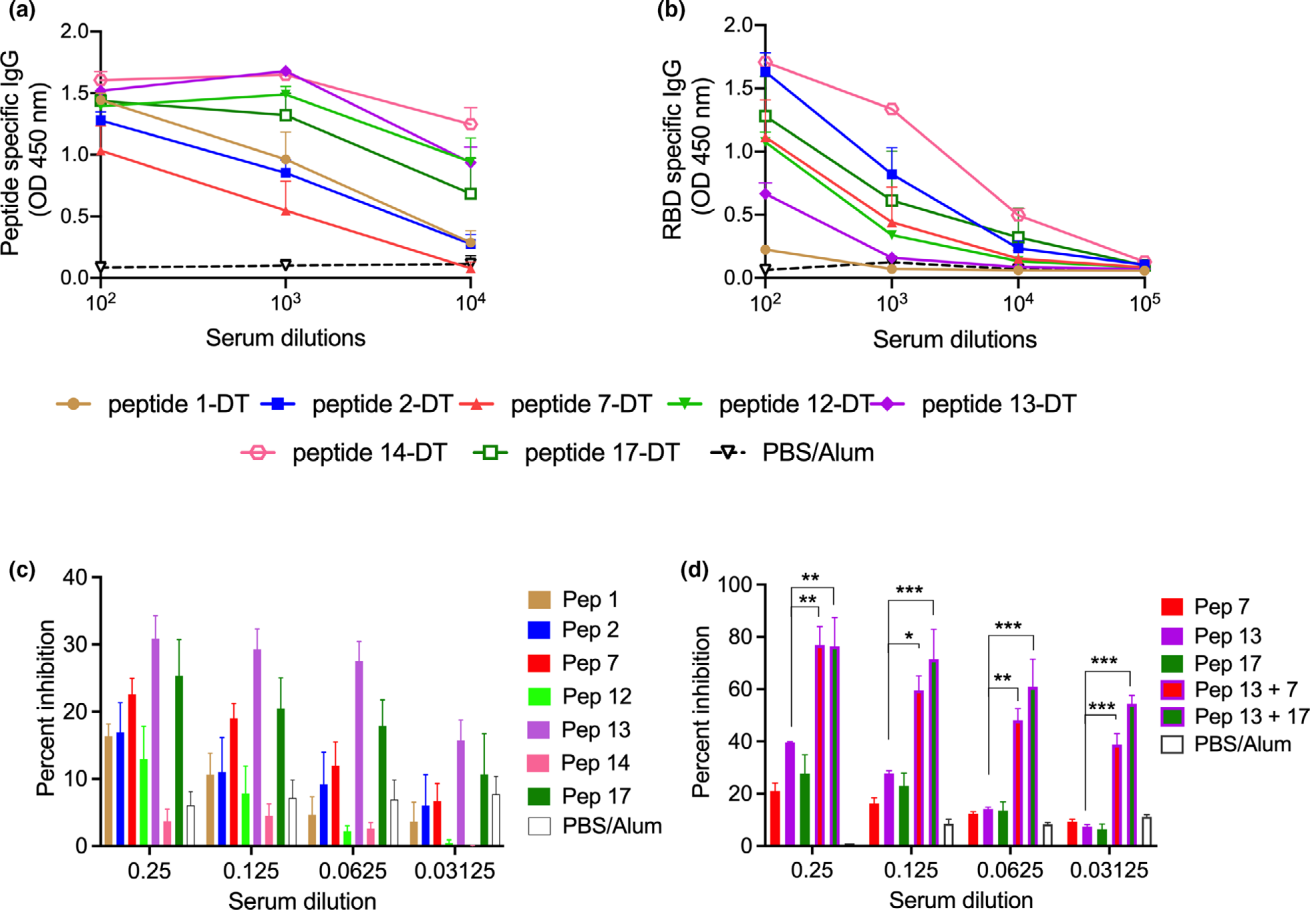
Specificity and function of peptide‐specific antibodies. **(a)** Peptide‐specific serum IgG. Mice (four per group) were immunised with peptide‐DT conjugates formulated in Alum. Seven days following three intramuscular immunisations, peptide‐specific serum IgG were assessed by ELISA. The data are Mean ± SEM. **(b)** Recognition of recRBD by peptide‐DT conjugate antisera. Sera were tested for their IgG binding to immobilised recRBD. **(c)** Inhibition of ACE‐2 and RBD binding by peptide antisera. Twofold serial dilutions of peptide antisera were added to immobilised recRBD; then, ACE‐2 conjugated to IgG‐Fc was added to each well and its binding to recRBD was detected as described in the Methods. Sera from PBS‐immunised mice were used as control. **(d)** Inhibition of ACE‐2 and RBD binding by individual or combination peptide antisera. Selected sera were then tested either alone or in combination for their inhibitory activity. Data are Mean ± SEM. Statistical analysis was performed using one‐way ANOVA with Tukey's multiple comparison test where mean inhibition caused by each peptide antisera was compared against mean inhibition by every other peptide antisera. Data for anti‐peptide 13 sera in comparison with combination peptide antisera (13 + 7 and 13 + 17), at various serum dilutions, are shown. *P < 0.05, **P < 0.01 and ***P < 0.001.

Each of the seven peptides was chemically conjugated to DT and formulated in Alum for studies in mice. Groups of BALB/c female mice were vaccinated intramuscularly with 25 μg of each peptide‐DT conjugate on days 0, 21 and 28. Sera were collected 7 days after the final boost and tested for their ability to recognise biotinylated peptides immobilised on streptavidin plates. All conjugates were immunogenic with peptide‐specific antibody titres reaching at least 10 000 (Figure 3a). All antisera, except for peptide 1–specific antiserum, also recognised recRBD (Figure 3b). Three antisera (to peptides 2, 14 and 17) recognised the RBD at a titre > 10 000. The peptide‐14 conjugate was the most immunogenic with an endpoint titre approaching 100 000. Peptide‐14 was also recognised by 38% of the CP and < 20% of NHS suggesting that it was immunogenic in many people as a result of infection with SARS‐CoV‐2. In all vaccinated cohorts, the level of RBD‐specific IgG at 3‐month post‐final vaccination was comparable to the levels seen at one‐week post‐final immunisation (Supplementary figure 5).

To determine whether the peptide‐specific antibodies were functional, we used a pseudo neutralisation assay^33^ and asked whether the sera could block binding of ACE2 to recRBD. We observed that antisera to peptides‐7, ‐13 and ‐17 caused partial inhibition of ~ 25% (Figure 3c). Antisera to peptide‐13 demonstrated the greatest inhibition. Peptide 14 antisera, which had the highest titre to recRBD (Figure 3b), had no functional activity. When we combined the antisera to peptide‐13 with antisera to either peptide‐7 or ‐17, we observed significant enhancement of inhibition in comparison with each of the individual peptide antisera (*P* < 0.05–0.001). The combination of antisera from peptide conjugates‐13 and ‐17 resulted in ~ 80% inhibition at the lowest dilution and ~ 58% inhibition at a dilution of 1:32. At that dilution, the individual antisera provided no inhibition (Figure 3d). Antisera to peptides‐7 and ‐13 also demonstrated significant synergistic activity.

### *Ex vivo* characterisation of SARS‐CoV2 peptide‐specific memory B‐cells (MBCs) in convalescent PBMCs

**Figure 4:**
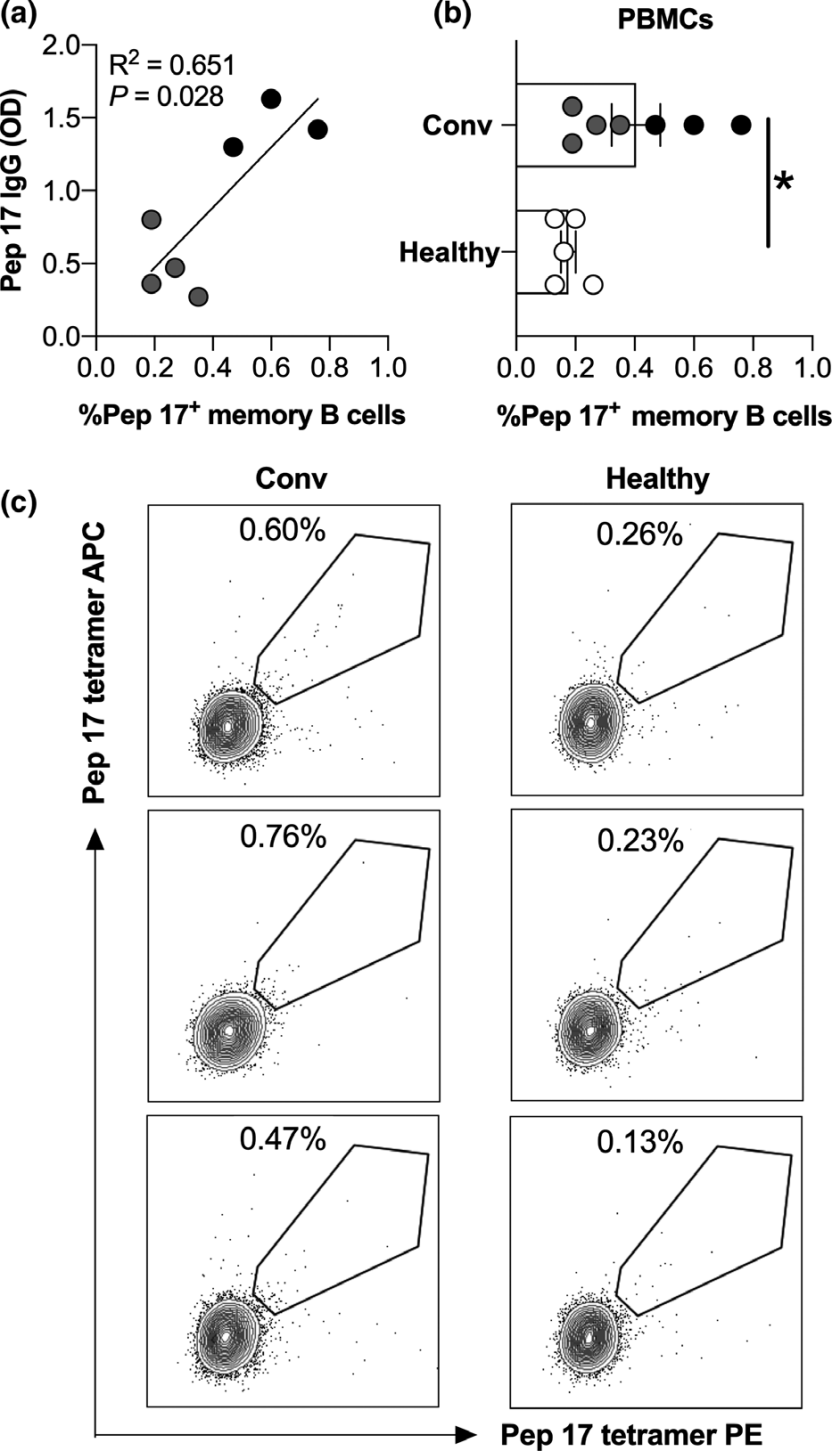
Peptide‐tetramer staining for memory B cells. **(a)** Peptide 17 tetramer staining of memory B cells (assessed as CD19^+^CD20^+^CD27^+^IgD^−^ cells) in healthy donors (healthy, *n* = 5) and COVID‐19 convalescent patients (Conv, *n* = 7). Black dots represent patients with high serum IgG titres (OD > 1), and grey dots represent patients with low serum IgG titres. *P < 0.05. Non‐parametric *t*‐test. **(b)** Correlation between peptide 17 (Pep 17) IgG in plasma and peptide 17 tetramer^+^ memory B cells in PBMCs of COVID‐19 convalescent patients. **(c)** Representative dot‐plot showing frequency of cells positive for peptide 17 tetramer in the memory B‐cell population of healthy donors and COVID‐9 convalescent patients.

To determine whether the three peptides were recognised by MBCs (CD3^−^ CD20^+^ CD19^+^ IgD^−^CD27^+^) in the convalescent patients, we prepared tetramers to peptides‐7, ‐13 and ‐17 and tested B‐cells from available PBMCs collected from seven of the convalescent patients (see Methods). The number of MBCs correlated directly with the level of peptide‐17‐specific IgG in CP (Figure 4a), and we found that MBCs that recognised peptide‐17 were significantly enriched in convalescent PBMCs in relation to healthy PBMCs (Figure 4b and c). No difference between the healthy and the seven convalescent patients was noted for peptide‐7 and peptide‐13 tetramer^+^ MBCs (not shown); however, these seven convalescent patients had low levels of peptide‐7 and ‐13‐specific antibodies.

## Discussion

We have identified linear epitopes on the RBD of the Spike protein of SARS‐CoV‐2 that are recognised by COVID‐19 CP and peripheral blood MBCs. We used different assays to identify seven candidate vaccine peptides, which were then chemically ligated to diphtheria toxoid and used to vaccinate mice. Peptide antisera were tested individually and in combination for their ability to inhibit RBD–ACE2 interaction. We observed significant synergism between different antisera suggesting that a vaccine containing two epitopes, either individually conjugated to a carrier protein and presented in the same formulation or presented on the same carrier protein molecule, could form the basis of a defined epitope‐based subunit vaccine.

Different strategies were used to identify the peptide epitopes. We initially thought that linear epitopes could be defined using an ELISA assay with peptides immobilised on the surface using the biotin‐streptavidin interaction. This assay identified certain epitopes recognised more strongly by CP than pre‐COVID normal human serum (#12, 13, 14 and 17). It also identified epitope #2, which was recognised by ~ 60% of both CP and pre‐COVID serum. To test for recognition of peptides in solution, where different epitopes may be accessible to antibodies, we used a competition ELISA and selected eight high titre CP. This assay identified peptide‐7, which was not recognised when immobilised on the plate, as well as the peptides recognised in the direct binding assay. Based on these data and the availability of reagents, we chose seven peptides for further investigation.

**Figure 5:**
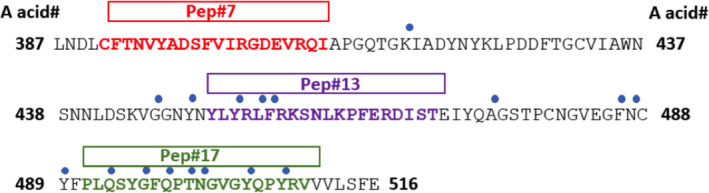
Contact residues between the RBD and ACE2 and positioning of selected peptides. Amino acid sequence of SARS‐CoV‐2 RBD with amino acid residue numbers (in black and bold) at each end of the sequence are depicted. The blue dots represent the contact residues, and the bolded amino acid represent the RBD peptide sequence for each of the three selected peptides. Adapted from Lan *et al*.^31^

Following conjugation and vaccination, three peptides (#2, #14 and #17) induced the highest titre antibody responses to recRBD. However, when tested for their ability to block the interaction of the RBD and ACE2, antisera to peptides 2 and 14 demonstrated low blocking activity, while antisera to peptides 7, 13 and 17 demonstrated the highest activity. Peptides‐7, ‐13 and ‐17 are not in regions shown to contain significant homology to human proteins.^25^ Peptides 13 and 17 contain three and seven known contact points, respectively, for interaction between the RBD and ACE2.^31^ The two peptides, which are not contiguous on the protein, represent the two blocks which contain most contact residues on the RBD (Figure 5). Interestingly, these contact residues are conserved between SARS‐CoV‐1 and SARS‐CoV‐2. The ability of antisera to an individual epitope to only partially block the binding suggests that when one block of contact points is bound by antibody, the other block of contact points permits sufficient interaction to allow binding. This would explain why when both are blocked (by antibodies to peptides 13 and 17 acting together) there is a synergistic increase in inhibition. It is not clear why antibodies to peptide 7, which does not contain any known contact residues, can block but it may be explained by steric hindrance by the large antibody molecule. Alternatively, antibodies to peptide 7 may distort the RBD and alter its ability to bind ACE2. It will be interesting to determine whether human vaccination with the entire Spike protein or mRNA encoding the entire protein will induce antibodies to these epitopes critical to RBD–ACE2 interaction and whether vaccine efficacy will depend on the induction of synergistic antibodies.

We noted that NHS contained antibodies to many peptides, although the fraction of peptides recognised and the extent of recognition was significantly less than the recognition by CP. We believe that this recognition by pre‐COVID‐19 serum represents the presence of antibodies to common cold coronaviruses which cross‐react with SARS‐CoV‐2. The sequence alignment of our RBD peptides with selected proteins from seasonal coronaviruses (*HCoV‐NL63, HCoV‐229E, HCoV‐OC43* and *HCoV‐HKU1*) has confirmed the homology between these sequences. We have not measured the affinity of these antibodies to the peptides; however, the pre‐COVID‐19 sera failed to recognise the recRBD, suggesting that their affinity is either low or that they arose because of exposure to other antigens. Whether they could provide any immunity to SARS‐CoV‐2 is unknown.

Polymorphisms within the Spike protein have been identified and will present a challenge to all vaccine programmes. However, Li *et al*.^34^ noted that while many mutations have been noted in the gene for the Spike protein, the mutation rate at any point is low. They tested 13 neutralising monoclonal antibodies (MAbs) against pseudo‐typed viruses representing natural variants and observed that antibodies to six distinct mutations within the RBD rendered the pseudo‐virus more resistant to the specific Mab. One was present in peptide‐13 (L452R) and another in peptide‐17 (Y508H). None were present in peptide‐7. Recently, a SARS‐CoV‐2 lineage, B.1.1.7, was reported to be spreading rapidly in the UK and is now found in several countries.^35^ It has a mutation within peptide‐17, N501Y, which is also a contact residue for RBD–ACE2 interaction. It is difficult to extrapolate to assess the impact that these mutations would have on the ability of polyclonal antibodies to block infection. However, it would be expected that polyclonal antibodies induced by peptide vaccination would target a cluster of related epitopes within the peptide span region and be less likely to be rendered ineffective by a single mutation. Of significance to vaccine development, the UK strain (B1.1.7), the South African strain (B1.351) and the Brazilian strain (P1) share a common mutation within Peptide #17, but are invariant within Peptide #7 and Peptide #13, compared to the original strain (CDC update, January 28, 2021).

We observed that SARS‐CoV‐2 infection led to the production of MBCs to a neutralising epitope within the RBD, defined by peptide‐17. Studies looking at MBCs to multiple epitopes on the RBD will lead to a better understanding of the nature and duration of natural immunity. Longevity of the immune response and immunological memory are also critical issues for a successful vaccine. We noted that antibody responses to the RBD following peptide vaccination did not diminish over 3 months. Ideally, a vaccine will provide life‐long immunity, but this may require intermittent boosting by exposure to the organism. The ability of infected individuals to generate peptide‐17‐specific MBCs suggests that infection would boost MBCs of this specificity in vaccinated individuals. Using identical peptide‐conjugate technology in a different system (streptococcus), we found that immunity was long‐lasting in mice and that re‐exposure of vaccinated mice to the organism could rapidly boost the number of antibody‐secreting cells and the antibody titre.^36^ This provided enduring protection from the organism. Given that example and the data presented in this paper, it is not unreasonable to expect that immune boosting by intermittent exposure to SARS‐CoV‐2 would occur post‐vaccination.

The level of inhibition of binding of the RBD and ACE2 required to protect from SARS‐CoV‐2 infection is not known. This could possibly be assessed by vaccinating and challenging human ACE2 transgenic mice; however, it can only be accurately determined following vaccination of volunteers in Phase III studies. We have used the human compatible adjuvant, Alum, but other human compatible adjuvants may induce higher antibody responses.

In summary, the data presented here demonstrate that SARS‐CoV‐2 infection leads to immunological memory and specific antibody responses that can be defined using synthetic peptides. Whilst antibodies to a single determinant provided limited inhibition of binding of RBD and ACE2, antibodies acting together to different parts of the molecule had significantly greater activity. Two of those regions contain the majority of contact points between the RBD and ACE2 and could be effective determinants in a defined epitope‐based vaccine. Vaccines may need to induce a bivalent antibody response. By limiting the number of epitopes in a vaccine to only two, the theoretical likelihood of inducing vaccine‐related immunopathology is reduced.

## Methods

### Ethics statement

We collected human samples under the human research ethics approval # HREC/20/QGC/63284 and (HREC/GU/2018/936). All animal procedures were conducted according to the Guidelines for the Care and Use of Laboratory Animals (National Health and Medical Research Council, Australia). Procedures were approved by the Griffith University Animal Ethics Committee (GUAEC).

### Human samples

Human plasma samples and PBMC were collected from COVID‐19 convalescent patients between February and May 2020 (after 3–4 weeks of recovery). Normal PBMCs, from healthy individuals, were collected between February and June 2020. All PBMCs were isolated using BD Vacutainer CPT (BD Biosciences, Franklin Lakes, NJ, USA) immediately following the blood collection and cryopreserved until analysis. Normal Human serum (NHS), collected pre‐COVID, were sourced from normal healthy individuals of an age range of 20–67 years, collected in Queensland in between 18/03/2019 and 04/06/2019 (HREC/GU/2018/936).

### Peptide library and proteins

For B‐cell epitope mapping with CP, the RBD sequence of SARS‐CoV‐2 (from amino acids 331–524 of the Spike protein) as previously described by Tian *et al*.,^37^ was utilised to design a synthetic peptide library as described in the Results section. Two sets of 19 peptides (each 20‐mer) overlapping by 10 amino acids were synthesised at Genscript, Piscataway, NJ, USA. One set was produced with N‐terminal biotin, and these peptides were used with streptavidin coated ELISA plates. Another set of peptides was made with C‐terminal cysteine to facilitate conjugation to the carrier protein, diphtheria toxoid (DT) for *in vivo* mouse studies. A random 20‐mer peptide was designed using a random number generator (LEKLEAQRRARDKSELKVDA) and was used as a negative control. The recombinant SARS‐CoV‐2 Spike protein (RBD, His‐Tag) encompassing amino acids Arg 319 ‐ Phe 541 (Accession # QHD43416.1) was sourced from Genscript and used in ELISAs. The recombinant SARS‐CoV‐2 (2019‐nCoV) Spike RBD‐His protein used in the ACE2‐binding inhibition assay was sourced from Sino Biological, Chesterbrook, PA, USA. Spike protein on virus‐like particles (Cspike on VLPs), used in SPR assay was produced using VSV system described below.

### Virus‐like‐particles

Non‐infectious lentivirus‐like particles were produced in the presence or absence of SARS‐CoV‐2 spike protein to mimic the presentation of SARS‐CoV‐2 Spike proteins on the surface of an enveloped virus. SARS‐CoV‐2 spike protein mammalian cell expression vector was a kind gift from Prof Linfa Wang at Duke‐NUS Medical School, Singapore. Production of VLPs and SARS‐CoV‐2 Spike pseudotyped VLPs were done by PEI mediated transfection of plasmid DNA into HEK 293 cells, and purification of these VLPs were done using virus purification procedures that we have previously described for HIV.^38^, ^39^

### Peptide synthesis and conjugation

Seven Cysteinyl peptides derived from the RBD peptide library were selected for conjugation to DT using 6‐Maleimido‐caproyl *n*‐hydroxy succinimide (MCS) chemistry as previously described ^40^. DT was produced at Olymvax Biopharmaceuticals, Chengdu, China.

### Microneutralisation of SARS‐CoV2 infection by convalescent plasma

The SARS‐CoV2 virus (SARS‐CoV‐2/CANADA/VIDO 01/2020) was obtained from the University of Saskatchewan, Canada (a kind gift from Dr. Darryl Falzarano). SARS‐CoV‐2 was produced by infection of Vero‐E6 cells. The amount of virus in stocks is determined by performing plaque assays at multiple dilutions and is expressed as pfu mL^−1^. N_100_ neutralising titres are determined in 96‐well plates in duplicate wells for each CP by a derivative of the TCID50 method.^41^ CP is diluted 1:15 in DMEM containing 2% FBS in the top row of a 96‐well plate, and then two fold serially diluted down the rows of the plate, followed by addition of an equal volume of media containing 100 pfu of SARS‐CoV2 (yielding a 1:30 dilution in the top well), and incubated for 1 h at 37°C. Vero E6 cells are infected with this mixture for 1 h at 37°C, and then the infection media is replaced by DMEM containing 2% FBS and the identical dilution of CP (1:30 in the top row), and further incubated. After 3 days, cells are fixed in 10% formaldehyde, and stained with 0.5% (w/v) crystal violet. The last dilution where there is an intact monolayer of cells is the N_100_ titre.

### Plaque reduction assay for convalescent plasma

Plaque reduction assays were conducted essentially as previously described.^42^

### Quantitation of virus‐specific IgG, IgA and IgM levels in convalescent plasma

Vero E6 cells in two identical 96‐well plates (one for IgG and IgA quantitation and one for IgG and IgM quantitation) were infected with MOI = 1 SARS‐CoV2 for 24 h, fixed with 4% formaldehyde, permeabilised with 1% Triton x‐100, blocked and then incubated with 1:1000 CP and antibodies specific for dsRNA (J2‐Sigma, St. Louis, MO, USA). One plate was stained with goat anti‐human IgA Dylight 594 (Thermo Fischer), goat anti‐human IgG Dylight 488 (Thermo Fischer, Markam, ON, Canada), and goat anti‐mouse Alexa 647 (Abcam, Cambridge, MA, USA) while the other was stained with goat anti‐human IgM Dylight 650 (Thermo Fischer), goat anti‐human IgG Dylight 488, and goat anti‐mouse Alexa 647 (Abcam). Nuclei were stained using DAPI. A total of 16 images for each duplicate well were taken using a Molecular Devices MetaXpress XLS widefield high content imaging system using the 40× objective, and the cell segmentation and fluorescence quantitation was done using MetaXpress software Molecular Devices, San Hose, CA, USA). The proportion of cells infected was approximately 75% as determined by dsRNA staining. In duplicate wells stained with each patient plasma, the average background fluorescence of uninfected cells was subtracted from each infected cell and then normalised using the dsRNA staining in that cell; and then the average normalised fluorescence was calculated for that CP. To validate the assay, we compared the levels of IgG, IgA or IgM fluorescence in stained cells to the dsRNA fluorescence. We also compared the normalised levels of IgG determined on the two identical plates and the normalised IgG, IgA and IgM levels to each other. Sample images of either NHS or CP staining on the IgG/IgA plate and the IgG/IgM plate are shown in Supplementary figure 2a and b.

### Surface plasmon resonance assay

recRBD or Spike protein on virus like particle (CSpike on VLPs) was immobilised on a high capacity Carboxymethyl dextran 3‐D Hydrogel surface biosensor (CDH) chip diluted at 20 µg mL^−1^ in sodium acetate pH 4.0. recRBD was amine coupled to the chip using an EDC/NHS activation of the dextran layer. The protein was flowed at 10 µL per min for 10 min to achieve an immobilisation level of between 600 and 1000 response units. An ethanolamine quenched flow cell is used as a control. To determine an antibody titre, serum was diluted in PBS running buffer (1:100–1:12 800+ in 1:2‐1:5 increments), run over the recRBD at 30 µL min^−1^ for 60 s and allowed to dissociate for 60 s prior to regeneration of the surface with a low sodium chloride buffer and pH change. To determine whether immunogenic sera/CP could inhibit the interaction between ACE2 and the recRBD, a competition assay between ACE2 and CP/spike protein were performed essentially as described.^43^ All SPR sensorgrams are collected using the ForteBio Pioneer software package and analysed using the QDat analysis package (ForteBio, Fremont, CA, USA).

### Tetramer staining for peptide‐specific memory B‐cells

*Ex‐vivo* characterisation of memory B‐cells (MBCs) was performed using antigen tetramers as previously described.^44^ Biotinylated peptides synthesised at Genscript were tetramerised by serial addition of premium‐grade streptavidin‐PE and streptavidin‐APC (Bio legend, San Diego, CA, USA) at a four‐molar equivalent of biotinylated peptide to one molar equivalent of streptavidin during 2 h at room temperature. The prepared tetramer was diluted to a concentration of 2 μm for use. Typically, 10^6^ PBMCs were pre‐incubated with Fc block (Bio legend) for 10 min and room temperature followed by tetramer staining on ice for 40 min. Cells were washed with PBS and then stained with a mixture of Abs for 30 min on ice: LIVE/DEAD Fixable Near‐IR (Molecular Probes, Waltham, Massachusetts, USA), anti‐CD3–FITC for T cell dumping (clone UCHT1; BD Biosciences) anti‐CD19–PerCP (clone HIB19; Bio legend); anti‐CD20–Alexa Fluor 700 (clone 2H7; Bio Legend), anti‐CD27–eFluor450 (clone O323; Molecular Probes) and anti‐IgD PE/Dazzle 595 (clone IA6; Bio legend). B‐cells were gated sequentially: lymphocytes (forward and side scatter), single cells, viable (NiRneg/CD3neg), and then CD20^+^CD19^+^IgD‐CD27^+^tetramerPE+tetramerAPC^+^ B cells for further analyses. BD LSR Fortessa (Griffith University) was used for cell acquisition. Data were analysed using FlowJo software (TreeStar, Ashlan, Oregon, USA).

### Immunisation and sample collection

BALB/c mice (female, 4–6 weeks) were immunised intramuscularly on day 0, 21 and 28 with 25 μg of peptide‐DT conjugates formulated with Alum adjuvant (2% Alhydrogel; InvivoGen, San Diego, CA, USA) in 1:1 ratio (v/v), for a total volume of 50 µL per mouse.^45^ Control mice received PBS with adjuvant alone.

### Enzyme‐linked immunosorbent assay

Indirect ELISA was used to determine antigen‐specific antibody levels in murine and human serum samples as previously described elsewhere.^30^ For recRBD‐specific ELISAs, recRBD (Genscript) was coated onto microtitre MaxiSorp plates (Thermo Fisher, Waltham, MA, USA) at a concentration of 1 μg mL^−1^. For peptide‐specific ELISA, streptavidin at 5 μg mL^−1^ (Sigma‐Aldrich) was first coated onto microtitre MaxiSorp plates followed by coating with biotinylated peptides at 1 μg mL^−1^. CP samples and mouse sera were both tested at various serial dilutions. Goat anti‐mouse IgG‐HRP at 1:3000 dilution (Bio‐Rad Laboratories, Hercules, CA, USA) was used to determine mouse IgG levels whereas goat anti‐human IgG‐HRP at 1:3000 dilution (Bio‐Rad Laboratories) or goat anti‐human IgM‐HRP at 1:10 000 dilution (Thermo Fisher, Hercules, CA, USA) was used to determine human antibody levels. SIGMA*FAST* OPD substrate (Sigma‐Aldrich) was added according to manufacturer's instructions and incubated at room temperature for 20 min.

### Competition ELISA

For competition ELISA assays, microtitre MaxiSorp plates were coated with 1 μg mL^−1^ of recRBD in 75 mm sodium carbonate buffer (pH 9.6) at 4°C overnight. Plates were washed with PBS containing 0.05% Tween 20 (PBST) and blocked for 90 min at 37°C with 5% skim milk/PBST. CP and NHS at 1:100 dilution were pre‐incubated with RBD‐derived peptides, recRBD (positive control) or control peptide (random sequence 20‐mer, negative control) at 100 μg mL^−1^ for 1 h at 4°C. Pre‐incubated sera were added to the blocked plates and incubated for 90 min at 37°C. The plates were washed four times with PBST, goat anti‐human IgG‐HRP at 1:300 dilution was added and plates were incubated for 90 min at 37°C. Plates were developed using SIGMA*FAST* OPD substrate (as above), and the absorbance was measured at 492 nm on a Tecan Infinite M200 Pro plate reader. Percent inhibition was defined by normalising the OD reading for the samples pre‐incubated with recRBD to 100% and the samples incubated with control peptide to 0%.

### ACE2‐binding inhibition assay

Serum samples were tested for anti‐RBD neutralising antibodies through ELISA, as previously described by He *et al*.^33^ Serum samples were tested for anti‐RBD neutralising antibodies through ELISA, as described by He *et al*.^33^ The equations for determination of inhibition are as follows:

To determine the content of bound or missing ACE2 in each well, the calibration curve (*n* = 4) was fitted to logistic function (*R* ^2^ > 0.98), Equation 1. The best fit equation was used with absorbance (*y*) values of each well to determine bound ACE2 content (*x*) by interpolation. Percentage of inhibition is determined from percentage of missing amount of ACE2, Equation 2.

(1) $$y=\frac{A_{1}‐A_{2}}{1+{\left(x/x_{o}\right)}^{p}}+A_{2}$$

(2) $$\mathrm{Inhibition}\%=\frac{{\mathrm{ACE}2}_{\mathrm{Total}}‐{\mathrm{ACE}2}_{\mathrm{Bound}}}{{\mathrm{ACE}2}_{\mathrm{Total}}}\times 100$$

where *y* is Absorbance readings, A1 and A2 are minimum and maximum absorbance readings, respectively, *x* is the amount of bound ACE2 concentration in ng/100 µL per well to be used in Equation 2, and *x*_0_ is the *x*‐axis (ACE2 concentration) centre point, p is the power exponent describes rate of change in absorbance signal with changing amount of bound ACE2, and ACE2 total is 200 ng/100 µL per well.

### Protein sequence alignment

Peptide sequences were aligned using BlastP protein sequence alignment tool from NCBI (https://blast.ncbi.nlm.nih.gov/Blast.cgi). All 19 peptides from the RBD peptide library were screened against the seasonal coronaviruses [Human Coronavirus HKU1 (taxid: 290028), Human Coronavirus OC43 (taxid: 31631), Human Coronavirus NL63 (taxid: 277944), Human Coronavirus 229E (taxid: 11137)]. Briefly, a standard blastP protein alignment search was performed for each of the 19 individual RBD peptides against four selected seasonal human coronaviruses. Positive alignments were then selected based on the ranking of the *e*‐value and max score and percent identity. Only alignments eliciting a significant low *e*‐value and high max score are represented in the associated peptide alignment data table.

### Statistical analysis

Data were analysed using GraphPad PRISM v8 (San Diego, CA, USA). Except where noted, data shown are means ± SEM. Statistical differences between the two groups were determined via the Mann–Whitney *U*‐test. For comparing multiple groups, one‐way ANOVA with Tukey's multiple comparison test was utilised. Correlation analyses were performed using Linear regression analysis with Pearson correlation coefficients used to define correlations between various parameters.

## Conflict of Interest

The authors declare no conflict of interest.

## Author contributions

**Manisha Pandey:** Conceptualization; Formal analysis; Funding acquisition; Investigation; Methodology; Project administration; Resources; Supervision; Writing‐original draft; Writing‐review & editing. **Victoria Ozberk:** Formal analysis; Investigation; Methodology; Project administration; Validation; Writing‐review & editing. **Sharareh Eskandari:** Formal analysis; Investigation; Methodology; Validation; Writing‐original draft; Writing‐review & editing. **Ahmed O. Shalash:** Formal analysis; Investigation; Methodology; Writing‐original draft. **Michael A. Joyce:** Formal analysis; Investigation; Methodology; Writing‐review & editing. **Holly A. Saffran:** Formal analysis; Methodology. **Christopher J. Day :** Formal analysis; Methodology. **Ailin Lepletier:** Formal analysis; Methodology; Writing‐review & editing. **Belinda L. Spillings:** Investigation; Methodology. **Jamie‐Lee Mills:** Investigation; Methodology. **Ainslie Calcutt:** Investigation; Methodology. **Fan Fan:** Funding acquisition. **James T. Williams:** Data curation. **Danielle I Stanisic:** Methodology. **Laetitia Hattingh:** Project administration; Resources; Writing‐review & editing. **John Gerrard:** Data curation; Resources; Writing‐review & editing. **Mariusz Skwarczynski:** Formal analysis; Investigation; Methodology. **Johnson Mak:** Investigation; Methodology. **Michael P. Jennings:** Data curation; Writing‐review & editing. **Istvan Toth:** Methodology; Writing‐review & editing. **D Lorne Tyrrell:** Data curation; Writing‐review & editing. **Michael F. Good:** Conceptualization; Data curation; Formal analysis; Funding acquisition; Investigation; Methodology; Project administration; Resources; Supervision; Validation; Writing‐review & editing.
