# Supplementary material for: Antibodies to neutralising epitopes synergistically block the interaction of the receptor‐binding domain of SARS‐CoV‐2 to ACE 2
Source: Clin Transl Immunology. 2021 Mar 7;10(3):e1260. doi: 10.1002/cti2.1260 (PMC7937407; doi:10.1002/cti2.1260)
Supplement: Supplementary file 1 [file CTI2-10-e1260-s001.pdf]

SUPPORTING INFORMATION

Supplementary Tables and Figures

Supplementary table 1. Participants details.

Participant no.	Gender	Age (years)	Symptomatic	Disease severity	Hospitalised?	Sample time-point (Days since recovery)
1	Male	66	Yes	mild	No	30
2	Female	29	Yes	mild	No	28
3	Male	61	Yes	mild	No	20
4	Male	55	Yes	mild	Yes	35
5	Female	74	Yes	mild	Yes	26
6	Male	77	Yes	mild	Yes	27
7	Male	60	Yes	mild	No	27
8	Female	64	Yes	mild	No	29
9	Male	36	Yes	mild	No	28
10	Female	78	No	mild	Yes - social reasons	32
11	Male	81	Yes	moderate	Yes	32
12	Male	67	Yes	mild	Yes	34
13	Female	67	Yes	mild	No	33
14	Female	31	Yes	mild	Yes	40
15	Male	50	Yes	mild	No	30
16	Male	58	Yes	mild	No	34
17	Female	61	Yes	severe	Yes	40
18	Male	63	Yes	mild	No	32
19	Female	60	Yes	mild	No	27
20	Female	49	Yes	mild	No	30
21	Female	27	Yes	mild	No	22
22	Male	30	Yes	mild	No	31
23	Female	81	Yes	mild	No	29
24	Female	35	Yes	mild	No	41
25	Male	57	Yes	moderate	Yes - 3x	44
26	Female	21	Yes	mild	No	23
27	Male	24	Yes	mild	No	29
28	Female	26	Yes	moderate	Yes	20
29	Male	37	No	mild	Yes (for quarantine purposes)	15-18
30	Male	43	No	moderate	Yes	15-18
31	Female	42	No	mild	Yes (for quarantine purposes)	15-18
32	Female	37	No	moderate	Yes	15-18

Supplementary table 2. Sequence alignment of RBD peptides and seasonal coronaviruses

RBD peptide	QUERY SEQUENCE	Percent identity	Max Score	Expect value	Alignment	Protein	Organism	Accession number
Peptide_1	NITNL**CPFGEVFNATRFASV**	52.94%	20.2	24	CPF--GEVFNATRFASV<br>CPF G V N F SV<br>CPFSFGKVINFVKFGSV	Spike protein	Human Coronavirus 229E	QEO75985.1
Peptide_1	NITNLC**PFGEV**FNATRFASV	100%	19.3	46	PFGEV<br>PFGEV<br>PFGEV	1a polyprotein	Human Coronavirus NL63	ABE73410.1
Peptide_2	VFNAT**RFASVY**AWNRKRISN	100%	22.7	3.1	RFASVY<br>RFASVY<br>RFASVY	replicase protein 1ab	Human Coronavirus OC43	QBP84756.1
Peptide_3	YA**WNRKRISNC**VADYSVLYN	66.67%	21.4	8.1	WNRKRISNC<br>W RK SNC<br>WERKIFSNC	spike protein	Human Coronavirus HKU1	ABU39969.1
Peptide_4	CVADYSV**LYNSASFS**TFKCY	75%	21.4	8.5	LYNSASFS<br>LYNS FS<br>LYNSSCFS	Hemagglutinin esterase	Human Coronavirus OC43	ARK08669.1
Peptide_5	SA**SFSTF**KCYGVSPTKLNDL	100%	18.9	66	SFSTF<br>SFSTF<br>SFSTF	Replicase polyprotein 1a	Human Coronavirus 229E	AGT21350.1
Peptide_6	GVSPTKL**NDLCFTN**VYADSF	85.71%	24	`1.1	NDLCFTN<br>NDLCF N<br>NDLCFAN	NSP4 (TM2	Human Coronavirus HKU1	YP_009944272.1

Peptide_7	C**FTNVYAD**SFVIRGDEVRQI	87.50%	22.3	4.3	FT-NVYAD<br>FT NVYAD<br>FTYNVYAD	Spike glycoprotein	Human Coronavirus HKU1	AGT17758.1
Peptide_7	CFTNV**YADSFVI**RGDEVRQI	71.43%	18.9	65	YADSFVI<br>Y DSF+I<br>YLDSFII	Non-structural protein 3	Human Coronavirus NL63	AFD64764.1
Peptide_8	**VIRGDEVRQIAP**GQTGKIAD	58%	19.3	47	VIRGDEVRQIAP<br>VI EV IAP<br>VI---EVKDIAP	ORF1a protein	229E-related bat coronavirus	APD51498.1
Peptide_9	APGQTGK**IADYNYKLP**DDFT	58.33%	22.3	4.3	IADYN---YKLP<br>I DYN Y LP<br>ITDYNYYKYNLP	RNA-dependent RNA-polymerase	Human Coronavirus HKU1	AXT92507.1
Peptide_10	**YNYK**LPDDFTGCVIAWNSNN	100%	18	129	YNYK<br>YNYK<br>YNYK	NSP6 (hydrophobic domain)	Human Coronavirus HKU1	YP_009944274.1
Peptide_10	**YNYKLPDDFTGC**VIAWNSNN	50%	18	130	YNYKLPDDF--TGC<br>Y YK DF GC<br>YYYKVEADFYLSGC	Hemagglutinin - esterase	Human Coronavirus OC43	AGT51480.1
Peptide_11	GCVIAWNS**NNLDSKV**GGNYN	75%	18.5	88	NNLD-SKV<br>NNLD SK+<br>NNLDKSKI	Spike protein	Human Coronavirus HKU1	ABU39971.1
Peptide_12	LDS**KVGGNYNYLYRLF**RKSN	53.33%	20.6	17	KVGGNYNYLYR--LF<br>KV GN+ Y +R LF<br>KVEGNFSY-FREELF	ORF1a protein	229E-related bat coronavirus	ALJ99893.1
Peptide_13	YLYR**LFRKSNL**KPFERDIST	70%	20.2	24	LFR---KSNL<br>LFR KSNL<br>LFRRKKKSNL	NSP15	Human Coronavirus HKU1	YP_460022.1
Peptide_14	LKP**FERDISTE**IYQAGSTPC	50%	17.2	255	FERDISTE<br>F+ ++STE<br>FDSEVSTE	Nucleocapsid	Human Coronavirus NL63	ABK63972.1

Peptide_15	**EIYQAGSTPCNGVEGFNCY**F	41.67%	22.7	3.1	EIY----- QAGSTPCNGVEGFNCY<br>E Y Q GS C FNCY<br>ELYVDFKPQSGSGTC- ----FNCY	Spike glycoprotein	229E-related bat coronavirus	ALK28781.1
Peptide_16	NGV**EGFNCY**FPLQSYGFQPT	66.67%	19.7	33	EGFN---CY<br>EGFN CY<br>EGFNILACY	Coronavirus main proteinase (3CLpro)	Human Coronavirus 229E	1P9S_A
Peptide_17	**PLQSYGFQPTNGVGYQP**YRV	52.62%	18.5	94	PLQSYGFQPTNGVGY- -QP<br>PLQ GF NGV + QP<br>PLQV-GF-- SNGVDFVAQP	replicase polyprotein 1ab	Human Coronavirus 229E	QOP39311.1
Peptide_18	NGVGYQPYRVVVL**SFELLH**A	83.33%	19.3	47	SFELLH<br>SFELL+<br>SFELLQ	Spike glycoprotein	Human Coronavirus NL63	QED88040.1
Peptide_19	VVL**SFELLH**APATV	83.33%	19.3	21	SFELLH<br>SFELL+<br>SFELLQ	Spike glycoprotein	Human Coronavirus NL63	QED88040.1

Amino acids in **bold** represent the amino acids that are shared between the query sequence and the protein of interest.

*identity. The extent to which two (nucleotide or amino acid) sequences have the same residues at the same positions in an alignment, often expressed as a percentage. (https://www.ncbi.nlm.nih.gov/books/NBK62051/).

**bit score (max score). The bit score, S', is derived from the raw alignment score, S, taking the statistical properties of the scoring system into account. Because bit scores are normalized with respect to the scoring system, they can be used to compare alignment scores from different searches.

***E value. The Expectation value or Expect value represents the number of different alignments with scores equivalent to or better than **S** that is expected to occur in a database search by chance. The lower the E value, the more significant the score and the alignment.

Supplementary figure 1

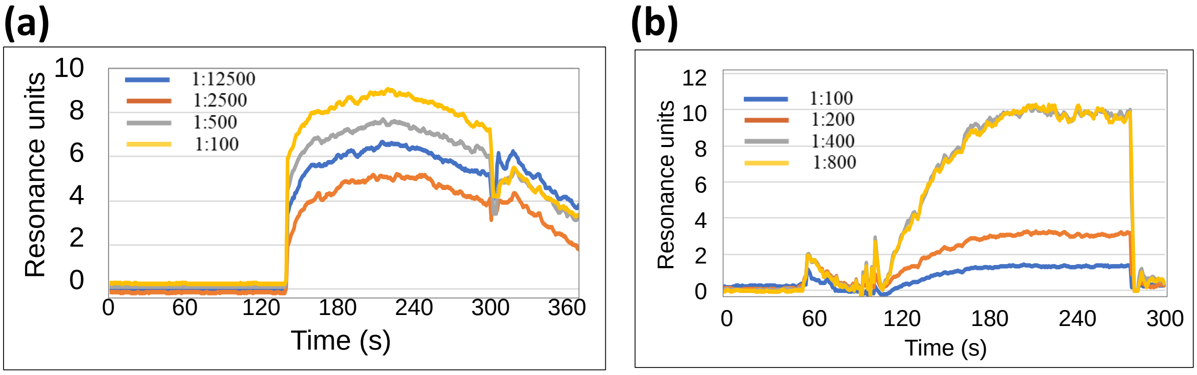

Supplementary figure 1. Binding to and neutralization of recRBD by CP. **(a-b)** *Surface Plasmon Resonance was performed using a Biocore T200 for affinity analysis*. recRBD or Spike protein on virus like particle (CSpike on VLP) is immobilized on a high capacity Carboxymethyl dextran 3-D Hydrogel surface biosensor (CDH) chip. recRBD was amine coupled to the chip using EDC/NHS activation of the dextran layer. CP at various dilutions was run over the protein and Ab binding to recRBD was determined **(a)**. To determine if CP could inhibit the interaction between ACE-2 and recRBD, competition assay was performed at various dilutions of sera and protein **(b)**. Representative images of each run are shown.

Supplementary figure 2

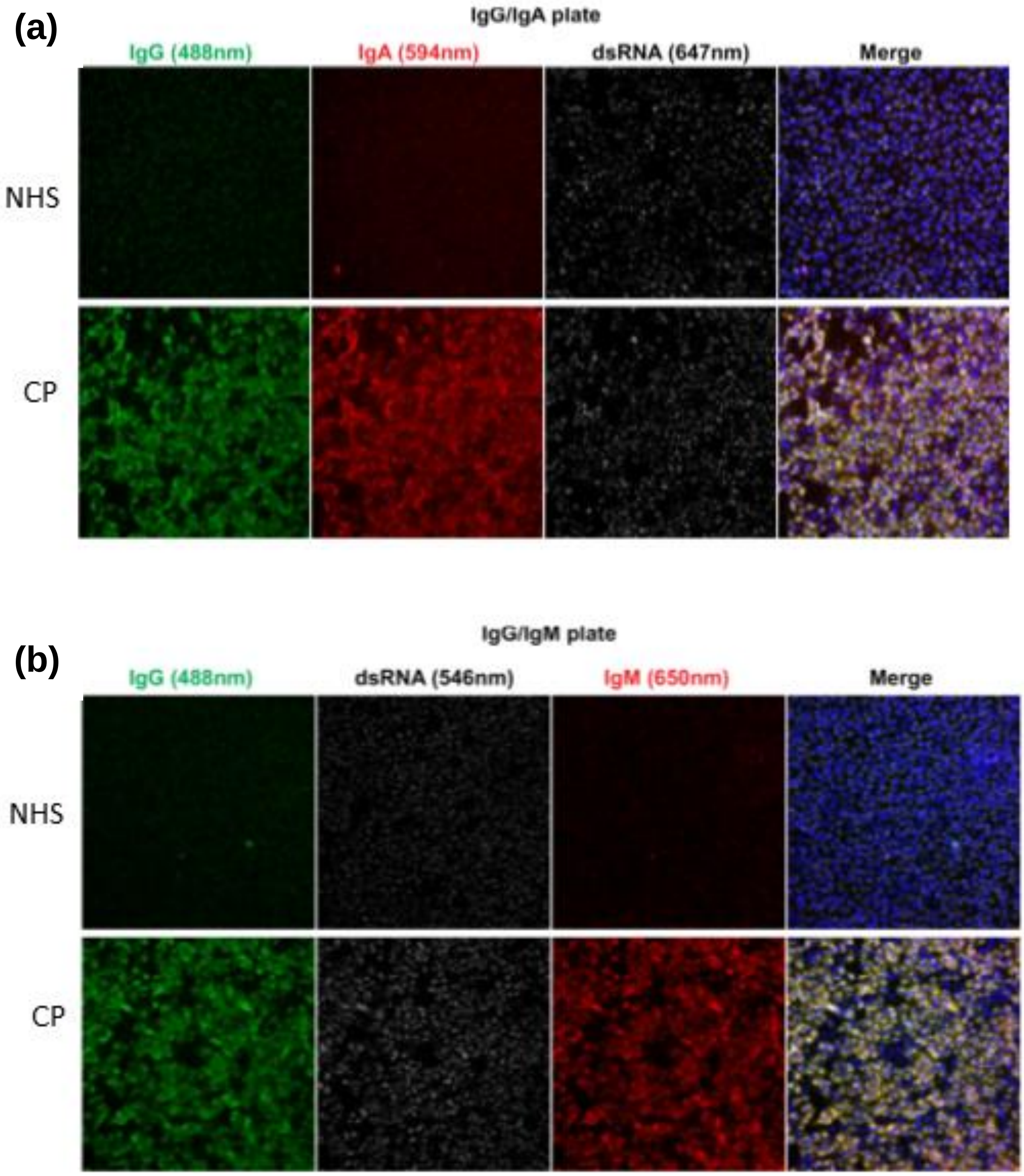

Supplementary figure 2. IgG, IgA and IgM staining and SARS-CoV. (a-b) Vero E6 cells in 2 identical 96 well plates (one for IgG and IgA quantitation and one for IgG and IgM quantitation) were infected with MOI=1 SARS-CoV2 for 24h, fixed with 4% formaldehyde, permeabilized with 1% Triton x-100, blocked and then incubated with 1:1000 CP and antibodies specific for dsRNA (J2-Sigma). One plate was stained with goat anti-human IgA Dylight 594 (Thermo Fischer), goat anti-human IgG Dylight 488 (Thermo Fischer), and goat anti-mouse Alexa 647 (Abcam) while the other was stained with goat anti-human IgM Dylight 650 (Thermo Fischer), goat anti-human IgG Dylight 488, and goat anti-mouse Alexa 647 (Abcam). Nuclei were stained using DAPI. Representative images of either NHS or CP staining on the IgG/IgA plate **(a)** and the IgG/IgM plate **(b)**. Wavelengths of the fluorophores are indicated.

Supplementary figure 3

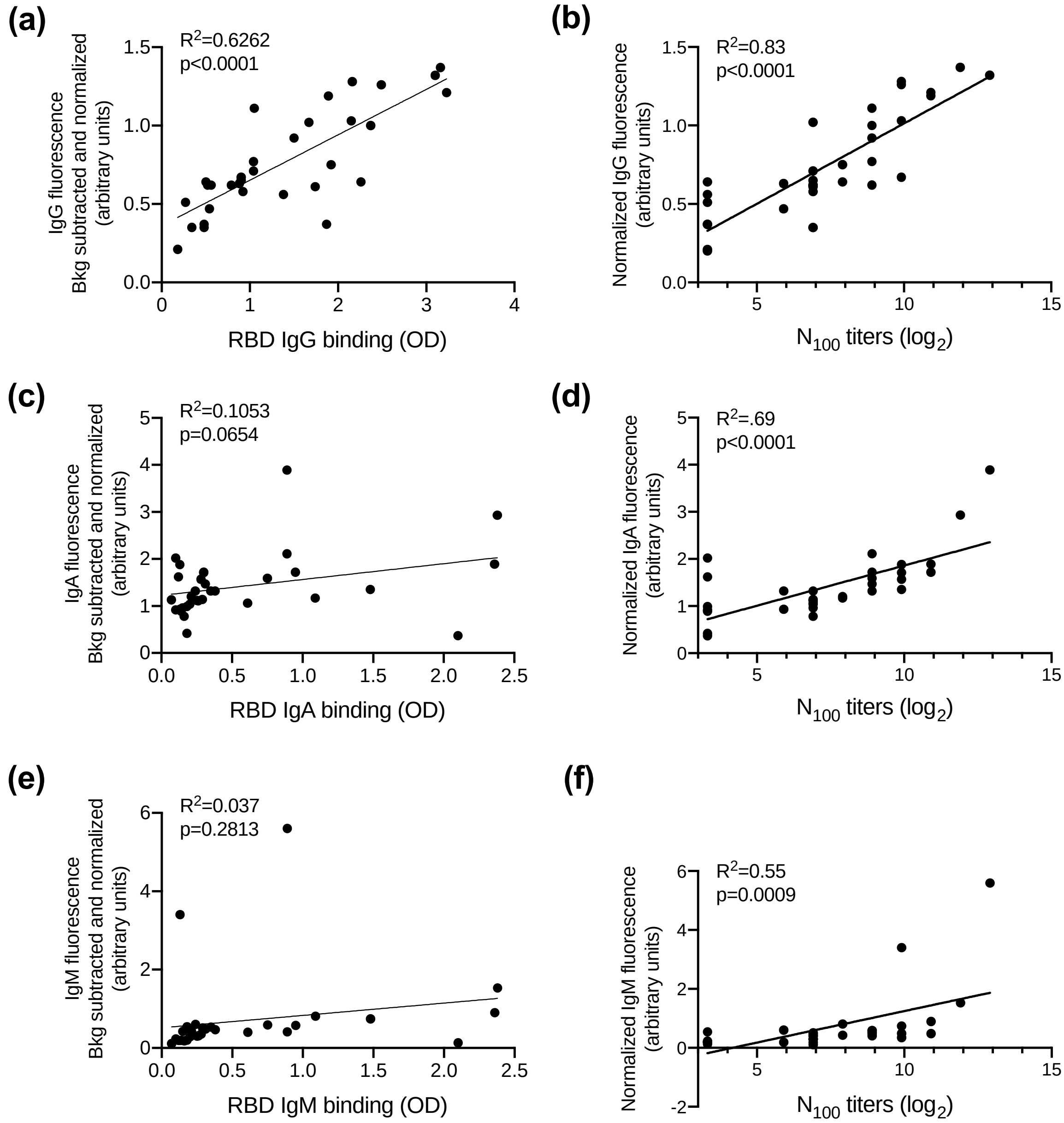

Supplementary figure 3. Correlations between SARS-CoV2 IgG, IgA or IgM levels in CP with either RBD binding or N_{100} antibody titers. The levels of SARS-CoV2 Ig subtype antibodies were measured and normalized by high content imaging of SARS-CoV2 infected cells stained with CP and visualized with anti-human IgG, IgA or IgM. The dsRNA antibody was used to monitor Vero cell infection. Unstained cells were subtracted as background and fluorescence was normalized to dsRNA staining on a per cell basis **(a, c and e)** as described in Methods; as are the N_{100} titers and the ability of CP to bind the RBD **(b, d and e)**. Linear regressions and Pearson correlation coefficients were calculated using GraphPad PRISM software.

Supplementary figure 4

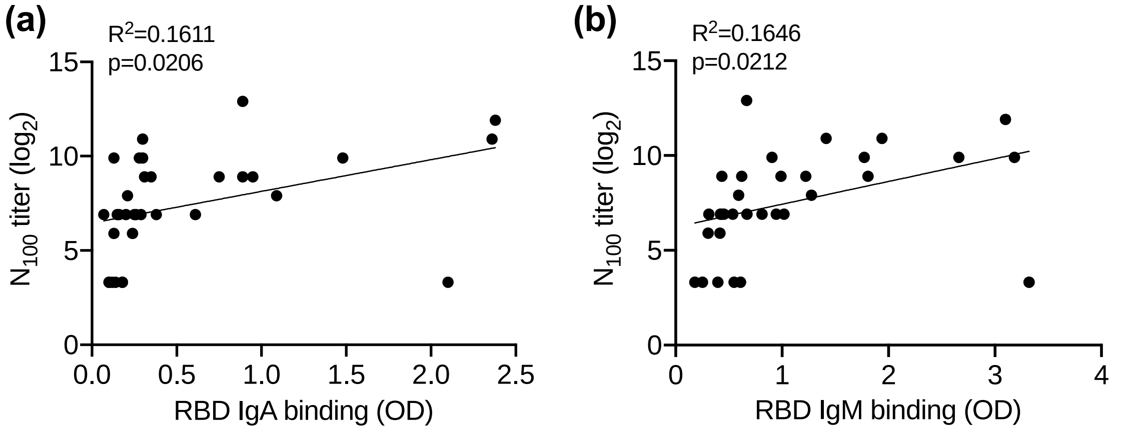

Supplementary figure 4. Correlation between RBD-specific IgA and IgM levels and virus neutralisation titer in CP. The levels of recRBD-specific IgA **(a)** and IgM **(b)** were measured in CP by ELISA. Linear regressions and Pearson correlation coefficients were calculated using GraphPad PRISM software.

Supplementary figure 5

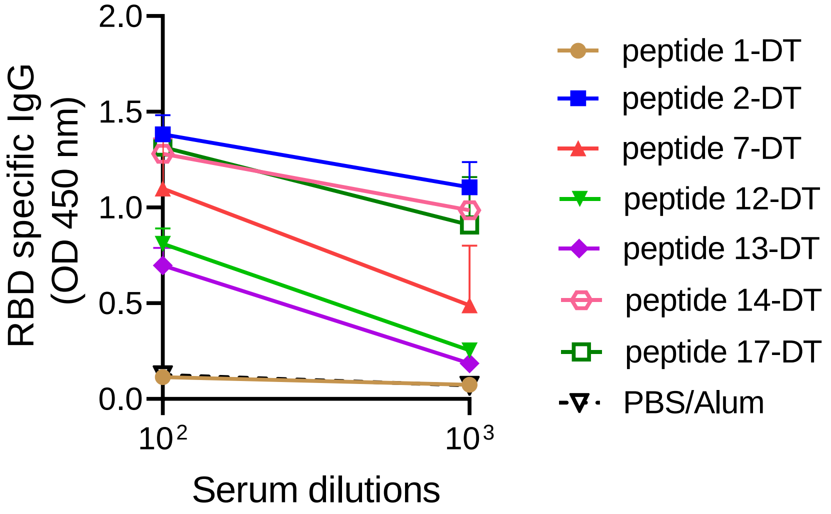

Supplementary figure 5. Recognition of recRBD by peptide-DT conjugate antisera at 3 months post immunisation. Mice (4 per group) were immunised with peptide-DT conjugates formulated in alum. Three-months post 3 intramuscular immunisations, RBD-specific serum IgG were assessed by ELISA. Sera were tested for their IgG binding to immobilised recRBD. The data are Mean ± SEM. Sera from PBS/Alum-immunised mice were used as control.

REFERENCES

1. Coll RC, Hill JR, Day CJ*, et al.* MCC950 directly targets the NLRP3 ATP-hydrolysis motif for inflammasome inhibition. *Nat Chem Biol* 2019; **15**: 556-559.
2. He Y, Lu H, Siddiqui P, Zhou Y, Jiang S. Receptor-binding domain of severe acute respiratory syndrome coronavirus spike protein contains multiple conformation-dependent epitopes that induce highly potent neutralizing antibodies. *J Immunol* 2005; **174**: 4908-4915.
